# Enhancing Learning in Outpatient Care Training: Theory Can Inform the Practice of Graduate Medical Education

**DOI:** 10.5334/pme.1576

**Published:** 2025-04-08

**Authors:** David C. Thomas, Janneke M. Frambach, Pim W. Teunissen, Frank W. J. M. Smeenk, Dario Torre

**Affiliations:** 1 Department of Medical Education, Department of Medicine and Department of Rehabilitation and Human Performance, Dean for Medical Education, Chair, Department of Medical Education, Icahn School of Medicine at Mount Sinai, New York, USA; 2 Department of Educational Development and Research, School of Health Professions Education (SHE), Maastricht University, Maastricht, the Netherlands; 3 School of Health Professions Education (SHE), Maastricht University, the Netherlands; 4 Department of Obstetrics & Gynecology, Maastricht University Medical Center, Maastricht, the Netherlands; 5 School of Health Professions Education (SHE), Maastricht University, Maastricht, the Netherlands; 6 Department of Medical Education, University of Central Florida College of Medicine, Orlando, USA

## Abstract

Caring for patients in the outpatient setting is a significant part of many physicians’ responsibilities due to healthcare’s shift from inpatient to outpatient settings. Outpatient care is complex and characterized by longitudinal relationships among all who work in this setting, including the patients. There is recognition of the need to enhance graduate medical education specifically situated in the outpatient setting. Considering that good educational practices need to be grounded in theoretical principles, the aim of this conceptual article is to analyze and explain learning in the outpatient care setting through a critical appraisal of selected learning theories. Four theories were selected to explore residents’ learning in relation to characteristics of the outpatient setting: 1) Cultural-Historical Activity Theory, 2) Situated Learning Theory, 3) Cognitive Development Theory and 4) Self-regulated Learning Theory. These theories were selected for their socio-cultural perspective or their focus on the learner. We highlight the implications for medical education and how these learning theories can inform teaching and learning in the outpatient care setting. For example, identification of contradictions and tensions between educational activity systems can promote expansive and transformational learning. By recognizing the unique opportunities for learning in the outpatient setting and applying learning theories, program directors and education specialists can develop better training programs resulting in more competent physicians to care and serve the needs of patients and society.

## Introduction

In recent decades we have seen a growth in outpatient care [1,2,3]. The move toward outpatient care is driven by multiple factors, including efforts to reduce inpatient admissions, to capitalize on cost-saving opportunities, the increase in outpatient procedures and to leverage of technological advancements. Subsequently, outpatient care has become a critical element of Graduate Medical Education (GME), serving as a cornerstone for training future physicians. Moreover, outpatient care will grow increasingly complex in the coming decades as older patients, with multiple chronic conditions, will outnumber younger, healthier individuals, challenging providers to manage a broader and more interconnected array of health issues. Globally, multiple chronic conditions occur in approximately one in three adults [4,5] and these adults utilize two to five times the number of physician visits as those without these conditions [5]. These shifts will require the need to enhance residents’ outpatient training for patients with chronic conditions and especially those with multimorbidity [6,7,8,9].

The field of outpatient care continues to see an increase in publications reflecting the recognition of its educational and clinical significance in modern healthcare systems [10]. We propose that the research in this field needs to be underpinned by appropriate theoretical lenses. The learning theories that inform educational practice applied to the inpatient setting may not simply be transferred to the outpatient setting. Good educational practices are grounded in and informed by sound theoretical principles [11]. Theory is described as “an abstract description of the relationships between concepts that help us to understand the world” and work in synergy to explain, interpret and further understand educational phenomena [11]. Elucidating the underlying theoretical frameworks of learning in outpatient educational practices may allow educators to enhance the quality and effectiveness of outpatient health professions education. In this conceptual paper, we examine selected theoretical frameworks for learning in outpatient care. We focus on physicians’ learning during their graduate medical education (GME) training. Previous literature examining the role of supervisors in General Practice emphasized the need for sharing of expectations for goals, tasks, and roles. Supervisors facilitate learning while establishing learning environments. More recent work builds on this to include the influences of environment, power, positioning, and agency [12,13,14,15]. The literature on learning in General Practice has drawn from a variety of learning theories and publications. Various learning theories argue that they could be applicable to General Practice and other outpatient care settings [16,17]. We posit that an exploration of selected learning theories in relation to the specific characteristics of outpatient care training, will enhance our understanding and potentially improve learning in and from outpatient training in GME. Learning theories can create a different way of observing and understanding the reality of outpatient training, enhance communication and scholarly conversations among medical educators, program directors, faculty, and residents. In sum, theoretical frameworks could offer directions for practical guidelines on how to optimize the outpatient clinical learning environment.

Outpatient care is described as providing medical services in an outpatient setting with no overnight stay in a hospital or inpatient facility [18]. We acknowledge that the international landscape consists of diverse settings including different types of healthcare system organizations, various types of providers and unique components in different regions of the globe. For this paper, we focus on two critical characteristics of outpatient education: 1) outpatient care as a complex system, 2) longitudinal relationships (resident-supervisor, resident-patient, patient-interprofessional team). As authors, our joint experiences in outpatient settings in graduate medical education, and our experiences of research and theories in medical education, led to the conception of this paper and have shaped its focus. Some authors are physicians and work in the outpatient care setting as both providers and supervisors in the Netherlands (FS, PT) and the USA (DCT, DT). All the authors have expertise in health professions education research. We recognize the strengths and limitations from drawing on our own experiences and contexts for shaping this paper. For example, it helped us to select learning theories that we feel are relevant to our outpatient care setting, yet this selection might not be the preferred one in other settings. We are cognizant that we are from high-income areas of the global community. Hence, our frame of reference leaned toward clinical settings with advanced technology and infrastructure. Our approach is from a constructivist stance routed in a socio-cultural perspective that promotes learning in context. Our premise is that individuals construct their learning, and the social context is an important factor for learning [19].

The aim of this conceptual paper is to analyze and explain how learning can be understood and may be enhanced in the outpatient care setting through selected learning theories [20]. We will first identify characteristics of outpatient care and training in this setting such as the complex system and longitudinal relationships. We will then discuss and explore learning and contextual characteristics of the outpatient setting through the lens of the selected learning theories. We chose the theories through a collaborative process and consensus building involving brainstorming and discussions recognizing we wanted to include theories from a sociocultural perspective and ones on how learning occurs within individuals. Although other theories could also be relevant, we believe the selected ones are particularly likely to offer a composite and valuable insight into teaching and learning within this setting from sociocultural and individual perspectives that can complement each other. This process resulted in the selection of the following theories: two focusing more on a sociocultural perspective, i.e., Cultural-Historical Activity Theory (CHAT), and Situated Learning Theory and two focusing on how learning occurs within individuals: Cognitive Development Theory and Self-regulated Learning Theory.

## Characteristics of the Outpatient Care Learning Context

### Outpatient care as a complex system

One could argue that the outpatient setting reflects a microcosm of the larger health care system, and as such, the primary characteristic is one of complexity, both at the macro- and micro-level. From the health system perspective (macro level) there may be competing objectives in outpatient care due to the hierarchical organizational structures [21]. Tensions between the needs of the organization and the needs of the learner are present in each encounter. For instance, the medical director may be concerned with productivity while the residents and supervisors are concerned with the amount of time available for teaching and learning. In addition to the organizational complexity, the actual resident-patient interaction in the clinic occurs within this complex system at the micro-level. Encounters in outpatient care are time limited, often hindering thorough investigation of patients’ social complexities and medical multimorbidity [22]. Decision making with limited time and data during the visit may cause the clinician concern, irrespective of the technologic explosion occurring in healthcare [23]. This requires a tolerance of ambiguity and uncertainty in both patient care and the resident’s supervision and learning.

### Longitudinal relationships (resident-supervisor, resident-patient, interprofessional team-patient)

The resident-patient and resident-supervisor relationships are crucial in outpatient care. The resident-patient’s central role involving interactions ideally fosters a therapeutic alliance through continuous, long-term engagement. Similarly, over time, the resident and supervisor develop increasing levels of trust during their interactions together [24]. Effective communication among the patient, resident, and interprofessional team influences patient experiences and outcomes. Importantly, all these interactions occur within the dynamic, interconnected, and non-linear complexity of the outpatient care system, requiring residents to adeptly manage real-time challenges and navigate hierarchical dynamics while coordinating care across various healthcare interactions [25]. Typically, these interactions occur over relatively long periods with multiple outpatient visits over time allowing them to build a trusting relationship [24,26,27]. Additionally, communication between the patient and interprofessional team may affect the patient experience and outcomes [21]. With many communities of health professionals interacting in the workplace, the role of hierarchy among individuals and groups may come into play. Hierarchies are pervasive in healthcare [28]. The desire is for these hierarchies to benefit patient care. However, the relationships also have implications for power struggles and communication [28]. Navigating these longitudinal relationships while providing patient-centered care in the outpatient setting requires residents to manage these relationship dynamics in real time [22].

Like the resident-patient relationship, the critical interaction that occurs between residents and their supervisors usually extends over longer periods of time, sometimes even years. The (dis)continuity in supervisors and the ‘quality’ of this supervisory relationship will affect residents’ learning. So, longitudinal relationships between the resident and supervisor exist and are important within the socio-cultural milieu of the learning environment [22]. As the individuals develop trust between them, especially over time, it allows residents to develop from novices to independent physicians. Over time, the supervisor can decide the level of supervision the resident requires which will span the gamut from a substantial amount early in training to very little by the end of training [24]. Understanding the characteristics of outpatient care, including the complexity of the health system and the unique attributes of individual learners, enables a deeper exploration of selected learning theories to ultimately enhance our understanding of learning in the outpatient setting.

## Applying Learning Theories to Outpatient Care

### Cultural-Historical Activity Theory

We selected this theory because of the insights it can provide into the characteristics of outpatient care in relation to residents’ learning, including outpatient care as a complex system and the longitudinal relationships between the resident, supervisor, patients and interprofessional team. Cultural-historical activity theory (CHAT), also known as activity theory, was described by Lev Vygotsky in the 1920’s and then expanded on by Alexei Leontiev. It is now in its third generation developed by Yrjö Engeström [29]. Activity theory provides a framework to examine complex interactions among people, rules, and tools, as well as the community, in order for a learner to attain a goal. According to this theory, human activity, and human learning, is goal-oriented with a desire to achieve specific outcomes within a given context. CHAT provides a comprehensive lens to analyze the sociocultural aspects of learning [30].

The current iteration of CHAT consists of five principles. The first principle is that activity theory takes the activity system as the primary unit of analysis [Bibr B31][Bibr B32]]. The activity system reflects human activity and learning. One system in outpatient care is the resident ‘providing care’ and is made up of its interconnected elements. These elements are the subjects (physicians, resident, nurses involved in the patient’s care), object (providing optimal care), tools and artifacts (electronic health records, instruments), rules (social norms of clinic, regulatory body oversight), community (residents, supervisors, health care providers, patients), and division of labor (roles of community members). These elements interact with each other in the clinical setting for the resident, together with other health care professionals, to provide care to patients. Here the second principle of activity theory becomes visible, which is the multi-voicedness of the activity system, emphasizing that the actors involved in the activity and the outpatient care community have multiple points of view and interests [31]. This relates to the third principle of activity theory, which is historicity: the activity system must be looked at with the lens of time where the system and its community is formed and transformed as the resident develops relationships with patients, supervisors and the healthcare team [31]. Multiple voices, opportunities and challenges are understood via a historical context and trajectory of the development of outpatient care in medicine.

**Figure 1:**
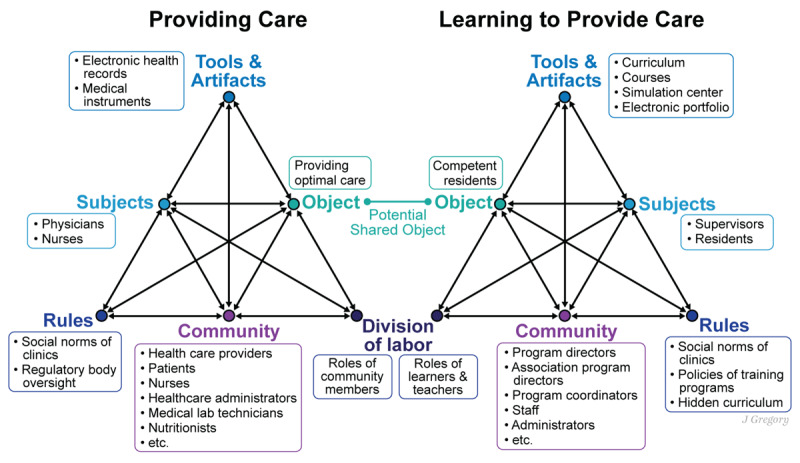
Based on Engeström’s activity triangle.

Before introducing the fourth and fifth principles, it is important to note that the activity system of ‘providing care’ is not the only one at play in the outpatient care training setting. A second interacting activity system is the resident ‘learning to provide care.’ The subjects remain the same (physicians, residents, other professionals) and the object is a competent resident. The tools and artifacts (curriculum, courses) interact with the rules (social norms and policies of training program, hidden curriculum), community (residents, teachers, medical students, and their collective stories), and division of labor (roles of learners and teachers in learning environment) (Figure 1). Juxtaposing these two activity systems highlights the fourth principle of activity theory, which is about the central role of contradictions as the driver of change and development [33]. Contradictions are not the same as challenges but rather systemic conflicts arising from the coexistence of opposing processes or goals within and between activity systems serving as catalysts for change and learning as the individuals and communities work to resolve them [31]. This is evident when looking at the resident “providing care” while “learning to provide care,” as some of the components in the two activity systems might be in contradiction with each other, resulting in tensions for the everyday experiences by the subjects. For example, as a learner, the resident may need more time for patient encounters than what is typically allowed in their role as a healthcare provider within the structure of outpatient care. Such contradictions and tensions lead to the fifth principle of activity theory, which emphasizes the possibility of expansive transformation of the activity and to transform into a next iteration [31]. The value of activity theory is that it brings these contradictions and tensions to the surface and allows the outpatient team to analyze them and identify what a transformation to the next iteration could look like. Activity theory can help identify learning tensions for resident’s activity systems and inform development of educational interventions. Educators may need to approach outpatient care teaching as a series of activity systems where interactions occur within a dynamic, interconnected setting in a non-linear manner, while also acknowledging the challenges of real-world implementation [34]. Residents and supervisors will need to adapt, manage real-time educational challenges and navigate ongoing tensions across different systems while simultaneously coordinating and delivering patient care [34,35,36].

### Situated learning theory and landscapes of practice

This sociocultural theory was selected as it emphasizes that learning occurs within a social context involving the communities where learning takes place [37]. Critical to the care of patients is collaboration with supervisors and members of the inter-professional team and the potential hierarchy involved in these encounters. Wenger and Lave originally described the concept of Communities of Practice (CoP) with three commonalities: mutual engagement, joint enterprise and shared repertoire [38]. Within CoP, the concept of domain represents the area of expertise around which the CoP forms and the residents develop these shared goals and knowledge with each other. While becoming a member of the CoP, the resident experiences mutual engagement while interacting and collaborating with other residents enabling the sharing of knowledge and contributing to learning. The notion of joint enterprise refers to the residents’ common purpose and goals which are developed in the shared practice. The residents furthermore have a shared repertoire of resources and tools such as the electronic health record and common language of patient care to deepen their sense of a community. The element of learning as participation enhances the process of the resident becoming more engaged in the community and contributes to additional knowledge and identity formation.

The CoP construct is further developed to encompass a recognition that the resident interacts with broader, interconnected communities of practice called the Landscapes of Practice (LoP). The LoP central tenets include identity formation and knowledgeability with the related notion of boundary crossing. The notion of boundary crossing requires the resident to intersect and move across boundaries of different CoPs. Enhancing opportunities for collaboration and breakdown of hierarchy occur during these interactions with different CoPs, i.e., supervisors and team members. The added component present in caring for patients includes working with other team members (i.e., an internal medicine resident discussing with an orthopedic consultant issues related to indications and contraindications for a procedure) that facilitates the resident’s knowledgeability and boundary crossing within these teams.

Lave and Wenger described ‘legitimate peripheral participation’ as being critical to identity formation. Legitimate peripheral participation is a social process of increasingly centripetal participation within a CoP resulting in the development of skill and professional identity. The longitudinal relationship with the supervisors and engagement in learning activities with a broader patient care team would be important to foster residents’ growing identity formation. Hence, the LoP provides the broader context in recognition of other communities, i.e., the interprofessional team, involved with learning [39].

Constructing the learning environment from a lens of the LoP requires the participation of all team members. Both supervisors and residents need to recognize learning opportunities to enable identity formation and boundary experiences. Promoting direct observation of residents’ interactions with other disciplines will allow educators to focus their teaching, provide supervision and engage learners across boundaries of LoP, facilitating the resident’s progressive development and independence. The LoP construct could align with a scheduling system in which the same group of residents rotate together for each outpatient care rotation over the length of their training. This journey through diverse practice landscapes offers residents new learning experiences as they navigate different communities, fostering the development of fresh perspectives in their learning.

The landscape of practice adds to our understanding of professional identity formation and the growth from novice to established physicians. The landscapes of practice highlight the importance of the relationship with the patient, supervisor and interprofessional team. The journey through landscapes of practice, each offering unique insights, challenges, and opportunities for growth fosters a dynamic and evolving process of identity formation [38].

### Cognitive development theory

We selected this theory as it highlights the importance of the longitudinal relationship between the resident and supervisor. This theory emphasizes the fundamental role of social interactions and culture as critical to learning. An important construct in Vygotsky’s cognitive development theory is the zone of proximal development (ZPD). It refers to the space between what a learner can do independently and what they need to master for additional learning. The ZPD consists of the area where the learning requires assistance or guidance, such as from a supervisor or an upper-level resident, to perform a task. These social interactions lead to the growth of the learner in the ZPD. The ZPD area contains tasks beyond the learner’s capabilities. Instruction is most beneficial in this zone. Scaffolding is an additional concept of the support and guidance provided in learning within the ZPD. As the residents’ competence increases over time the amount of scaffolding support decreases to allow for the growth of their independent knowledge and skills.

With the development of the resident over time these supports decrease to promote competence in the quest for independent practice in outpatient care. The opportunity for direct observation of the learner moving through multiple ZPDs allows for the monitoring of the progress and trajectory of the learner. Highlighting the characteristics of this longitudinal relationship and trust that develops, faculty can assist in identifying the current zone of proximal development, provide feedback, support and guidance to achieve the next ZPD, helping the resident progress toward independent practice.

### Self-regulated learning theory

This theory was selected as it continues to build on the important characteristic of outpatient care recognizing the skills needed for the resident to develop in this setting and the active role the resident plays in their learning. We will approach self-regulated learning (SRL) from the social cognitive perspective in line with a socio-cultural lens [40,41]. SRL focuses on the learner being actively engaged in and responsible for their own learning and therefore academic successes. This focus helps the learner to understand he motivation to learn as well as the intellectual and emotional stimulus for the learner to succeed. Individual, contextual and social factors influence SRL in the clinical learning environment [42]. Cyclical and linked phases of self-regulated learning help in understanding and guiding the learner and may be uniquely influenced by the type of clinical setting.

The forethought phase of SRL involves goal setting, strategic planning as well as self-motivation involving one’s goals and vision of outcomes. The performance phase includes learning with self-control and self-monitoring. The self-reflection phase requires self-evaluation as well as self-reaction identifying one’s satisfaction level with the outcome [43]. These phases highlight the active role that the learners have in their own learning. Self-efficacy, metacognition and goal setting are core principles at play with SRL and require the learner to be an active participant in their own learning process in the clinical learning environment of outpatient care [43].

In practice, learners can apply these SRL strategies in outpatient care with their interactions with patients, supervisors, and the healthcare team. Recognizing the affordances of the outpatient care environment (electronic health record, decision support systems, other health professionals, supervisors, colleagues) enables residents to identify and capitalize on the diverse learning opportunities available in this setting. These opportunities are then also present for all the members of the healthcare team in the learning environment. Self-assessment and reflection on their patient care skills will be important to identify areas of strength and more importantly areas for improvement in caring for patients in outpatient care. Notably, much of the responsibility for learning then lies with the residents and could be challenging for residents less engaged in wanting to learn in outpatient care. By encouraging residents to engage in self-regulated learning, faculty play a crucial role in fostering the development of the competence required to become proficient physicians in outpatient care allowing residents to take ownership of their own learning.

## Implications for Graduate Medical Education in Outpatient Settings

Attention to these learning theories enables one to capitalize on the affordance of the learning environment with special focus on both the socio-cultural and individual nature of learning. The selected learning theories can play a crucial role in shaping and improving the design of educational environments. Here, we highlight two examples of how these theories could be of use: interprofessional education and longitudinal clinical experiences.

Social interactions across disciplines invite different perspectives during care and learning and are important conditions for *inter-professional education*. Enabling learners to interact with other disciplines involved in patient care emphasizes the concept of the landscape of practice. Sustained interactions further develop the learners’ legitimate peripheral participation and professional identity formation. For example, although social workers are present in many settings, the longstanding interactions in outpatient care allow the resident to learn unique components of their role, like identifying outpatient resources and long-term coordination of care with the patient. Outpatient education may benefit from highlighting the importance of relationships (resident-supervisor-patient-team) within and among learning communities and systems and future research may explore which components of these longitudinal relationships enhance learning and identity formation.

Incorporating activity theory into examining the learning environment focuses on an appreciation of social interactions among all members of the community while identifying tensions that may result in learning [35]. In addition, activity theory enables identifying the tools, artifacts and rules specific to the activity system. Expansive learning is built upon the principles of CHAT. The primary goal of expansive learning is to promote change by transforming practices and systems to address existing contradictions and challenges. Expansive learning is characterized by cycles of learning actions that lead to the development of new practices, negotiating different perspectives through a collaborative process [44].

CHAT provides a lens to analyze the complexities and contradictions within healthcare systems. By recognizing these systemic issues, medical education can better prepare residents to navigate real-world challenges in healthcare settings [45,46]. It also emphasizes the interconnectedness of various activity systems within healthcare. Educators can leverage this understanding to foster collaboration and meaningful connections between educational contexts, healthcare systems, and communities, ultimately enhancing the quality of care [30,32].For example, the nutritionist develops plans to help support health and manage chronic conditions, the resident learns the tools used by them. The focus on inter-professional education is highlighted for the learner to develop their professional identity moving from observer to active participant with additional responsibilities all while working within the complex environment of outpatient care. Highlighting the need for highly functioning teams that provide optimum patient care may direct further research to identify what the characteristics of these teams are in outpatient care.

Residency programs continue to look for opportunities to develop robust *longitudinal clinical experiences*. Engaging in types of experiences with various disciplines promotes meaningful relationships that can lead to participation in LoPs resulting in relevant learning outcomes. Over time, supervisors would have more opportunities for meaningful interactions and conversations, allowing them to identify the residents’ zone of peripheral development and tailor their teaching to provide the appropriate level of challenge and support, fostering the resident’s growth. Interestingly, this longitudinal placement with the same supervisors and team members would highlight the activity systems at play for the resident. Curricula for learners in outpatient care settings could be designed to allow identification of the contradictions and tensions between ‘providing care’ and ‘learning to provide care’ thus also promoting transformation of the learning environment. Changes to outpatient care could involve a larger move of residency programs to have longitudinal clinical experiences for all residents while allowing researchers to investigate how to mitigate these tensions.

Theories play a crucial role in informing educational practice by providing a structured framework for decision-making in curriculum design, implementation, and assessment. By incorporating multiple and complimentary theories, health professions educators can ground their choices in well-established research, not only enhancing existing instructional strategies but also creating a research agenda to guide ongoing inquiry in outpatient education. We invite readers to think about the implications for future research in this area. How do the components of these theories shed light on optimal learning for residents? What component of the theory has the greatest impact on learning? We encourage others to explore this area and share their findings with the community.

Healthcare systems vary significantly across different contexts and cultures, with each system shaped by local resources, needs, and cultural norms. Our perspectives are rooted in North American and Western European high-resource countries with technology, infrastructure and cultural beliefs and practices, which may have influenced our insights. As a result, our views might not fully reflect the challenges and learning environments in other settings, where healthcare practices and educational needs could differ greatly. Recognizing this variability is essential for fostering a more comprehensive understanding of how healthcare systems and learning environments evolve in diverse contexts. Furthermore, by focusing on these four theories, we limited our exploration to specific theoretical perspectives. Considering additional theories might have introduced alternative viewpoints and led to different considerations, potentially broadening our understanding, and offering new insights into the issues at hand.

## Conclusion

The exploration of these theories elucidates important constructs useful in understanding learning in outpatient care. With this understanding, medical educators can develop curricula and assessments specific for the outpatient care environment. One will want to consider the context and culture of the learning environment during all phases of curricular design. Recognition of community and social interactions involvement in learning is essential in educational planning and assessment. Ultimately, the aim of considering learning in outpatient care with the lens of learning theories is to develop better training programs for residents, resulting in more competent physicians to provide care and serve the needs of society.
